# Morning Hypertension as a Factor Independently Associated with Renal Dysfunction in Hypertensive Patients with Chronic Kidney Disease

**DOI:** 10.3390/medicina62101911

**Published:** 2026-10-03

**Authors:** Samet Usta, Özdem Kavraz Tomar, Ertan Aydın, Numan Kılıç, Tülin Akagün

**Affiliations:** 1 Department of Internal Medicine, Giresun University Faculty of Medicine, Giresun 28200, Türkiye; sametuusta@gmail.com; 2 Division of Nephrology, Department of Internal Medicine, Giresun University Faculty of Medicine, Giresun 28200, Türkiye; tulinozbay@yahoo.com; 3 Department of Cardiology, Giresun University Faculty of Medicine, Giresun 28200, Türkiyedr.numanklc@gmail.com (N.K.)

**Keywords:** morning hypertension, chronic kidney disease, target organ damage, ambulatory blood pressure monitoring, nocturnal hypertension

## Abstract

*Background and Objectives*: Hypertension is highly prevalent among individuals with chronic kidney disease (CKD) and contributes substantially to both cardiovascular morbidity and progressive renal dysfunction. Ambulatory blood pressure monitoring (ABPM) provides superior diagnostic accuracy compared with office measurements and is recommended for evaluating blood pressure patterns and hypertension-mediated organ damage in CKD. Morning blood pressure, in particular, has gained increasing attention due to its association with adverse cardiovascular and renal outcomes. However, evidence regarding the independent impact of morning hypertension in CKD remains limited. This study aimed to determine the prevalence of morning and nocturnal hypertension phenotypes in hypertensive CKD patients and to evaluate their associations with renal and cardiac target organ damage. *Materials and Methods*: A total of 132 patients with CKD stages 1–5 underwent 24-h ABPM, transthoracic echocardiography, and comprehensive biochemical evaluation. Morning and nocturnal hypertension were defined according to ESC 2024 guidelines. Demographic, clinical, echocardiographic, and renal parameters were compared between groups. Correlation analyses were performed to explore associations between blood pressure phenotypes and markers of target organ damage. Multivariable regression models were used to assess independent predictors of renal and cardiac structural alterations. *Results*: Morning hypertension was present in 42.4% of patients, whereas nocturnal hypertension was detected in 78%. Patients with morning hypertension exhibited significantly lower eGFR and higher UACR levels (both *p* < 0.001). Morning hypertension showed positive correlations with creatinine, urea, PTH, ESR, and UACR, and negative correlations with eGFR, albumin, calcium, hemoglobin, and TAPSE. In multivariable regression, morning hypertension was independently associated with lower eGFR (β = −0.26; *p* = 0.002). Nocturnal hypertension was positively associated with IVSd (β = 0.19; *p* = 0.024) and UACR, supporting its role in cardiac remodeling and renal injury. *Conclusions*: Morning hypertension may represent a clinically relevant blood pressure phenotype in CKD and may be associated with markers of renal dysfunction; however, longitudinal studies are needed to determine its prognostic significance. The strong associations of nocturnal hypertension with IVSd and albuminuria further highlight the importance of evaluating 24-h blood pressure patterns. Incorporating ABPM into routine clinical practice may enhance risk stratification and guide individualized treatment strategies in CKD.

## 1. Introduction

Chronic kidney disease (CKD) is one of the clinical conditions most frequently accompanied by hypertension [1]. In patients with CKD, the presence of hypertension markedly accelerates both cardiovascular complications and the decline in kidney function [2,3]. Ambulatory blood pressure monitoring (ABPM) provides more accurate information than office measurements for assessing blood pressure variability and hypertension-related target organ damage, and is therefore recommended for the evaluation of patients with CKD [4,5]. Among ABPM parameters, morning blood pressure has gained increasing attention in recent years, and previous studies have demonstrated that morning hypertension is associated with cardiovascular events, left ventricular hypertrophy (LVH), urine albumin-to-creatinine ratio (UACR), and deterioration in kidney function [6,7,8].

Several mechanisms contribute to the development of morning hypertension, including increased sympathetic activity, insufficient nocturnal blood pressure decline, arterial stiffness, and reduced effectiveness of antihypertensive medications during the early morning hours [9,10]. These mechanisms become more pronounced in CKD, where autonomic dysfunction, volume overload, and vascular stiffness are common. Morning hypertension may contribute to chronic kidney disease progression through several interrelated mechanisms. Increased early-morning sympathetic activation, impaired nocturnal sodium excretion, endothelial dysfunction, heightened arterial stiffness, and disruption of circadian blood pressure regulation collectively create a pathophysiological environment that promotes both glomerular hyperfiltration and subsequent structural injury. These mechanisms may also reinforce each other, forming a vicious cycle in which elevated morning blood pressure accelerates renal damage, while declining kidney function further amplifies morning BP elevation. Clarifying these mechanistic links provides a stronger rationale for evaluating renal (eGFR, UACR) and cardiac (IVSd, TAPSE) target organ damage markers and for establishing correlational relationships between morning hypertension and organ injury [6,8,9]. Although studies in the general hypertensive population have shown an association between morning hypertension and target organ damage, evidence in CKD patients remains limited. Recent investigations have reported that morning hypertension is associated with LVH, increased left ventricular mass index (LVMI), UACR, and lower estimated glomerular filtration rate (eGFR), and that these associations may be independent of nocturnal hypertension or the morning blood pressure surge [6,8,11,12]. Nevertheless, the extent to which morning blood pressure regulation contributes to cardiac and renal structural alterations in CKD has not been fully elucidated.

Given the high cardiovascular morbidity and the progressive nature of kidney function decline in CKD, the identification of early markers of target organ damage is of substantial clinical importance. Clarifying the relationship between morning hypertension and indicators of cardiac and renal target organ damage may contribute to improved risk stratification and more precise treatment strategies. Furthermore, determining whether the effects of morning hypertension are independent of nocturnal or office hypertension may further refine approaches to blood pressure management in CKD. Therefore, this study aims to evaluate the association between morning blood pressure regulation and cardiac and renal target organ damage in hypertensive patients with chronic kidney disease, and to assess whether this relationship is independent of nocturnal and office hypertension.

## 2. Materials and Methods

### 2.1. Study Design and Participants

This prospective observational study included a total of 132 adult patients with a diagnosis of chronic kidney disease (CKD) who presented to the nephrology outpatient clinic of a tertiary care training and research hospital between October 2024 and June 2025.

Inclusion criteria were:

1. Age between 18 and 80 years;
2. Diagnosis of CKD stages 1–5;
3. Not receiving dialysis therapy;
4. Provision of informed consent.

Exclusion criteria included:

1. New York Heart Association (NYHA) class III–IV heart failure;
2. History of coronary artery bypass grafting;
3. Permanent pacemaker implantation;
4. Malignancy;
5. Pregnancy;
6. Previous kidney transplantation.

The diagnosis of CKD was established according to the Kidney Disease: Improving Global Outcomes (KDIGO) 2024 criteria, based on the presence of kidney damage markers such as albuminuria or an estimated glomerular filtration rate (eGFR) < 60 mL/min/1.73 m^2^ persisting for ≥3 months. eGFR was calculated using the Chronic Kidney Disease Epidemiology Collaboration (CKD-EPI) equation.

The definitions and classifications of hypertension, as well as the assessment of target organ damage, were based on the 2024 European Society of Cardiology Hypertension Guidelines (ESC 2024). Cardiac target-organ damage was evaluated using interventricular septal thickness (IVSd) as an indicator of left ventricular remodeling; LVH was not directly assessed because LV mass index was not measured. Renal target organ damage was evaluated using decreased eGFR and elevated urine albumin-to-creatinine ratio (UACR) values.

### 2.2. Clinical and Laboratory Data

The clinical evaluation of the patients was performed during their nephrology outpatient clinic visits. At presentation, demographic characteristics (age, sex), comorbidities (diabetes mellitus, cardiovascular disease, cerebrovascular disease), and current antihypertensive medications were recorded. Following clinical assessment, office blood pressure measurements were obtained using an automated brachial blood pressure device after the patients had rested for at least 5 min in accordance with standard protocols. Two measurements taken 3 min apart were averaged and documented. On the same day, laboratory analyses including hemoglobin, albumin, creatinine, eGFR, electrolytes, uric acid, parathyroid hormone (PTH), lipid profile, and urine albumin-to-creatinine ratio (UACR) were performed and the results were recorded. BMI, smoking status, and HbA1c levels were not included in the analysis because these variables were not consistently available for all participants, and inclusion of incomplete data could introduce bias.

### 2.3. Echocardiographic Assessment

After completion of office blood pressure measurements, transthoracic echocardiography (TTE) was performed using an EPIQ CVx ultrasound system (Philips, Eindhoven, The Netherlands) by two experienced cardiologists to evaluate cardiac target-organ damage. Left ventricular remodeling was assessed using end-diastolic interventricular septal thickness (IVSd), and values ≥ 1.2 cm were considered indicative of increased septal thickness in accordance with guideline-based thresholds. As part of the echocardiographic evaluation, left ventricular ejection fraction (LVEF), tricuspid annular plane systolic excursion (TAPSE), and pulmonary artery pressure (PAP) were measured. IVSd thresholds were defined according to ASE/EACVI echocardiography recommendations.

Right ventricular systolic function was assessed using TAPSE, measured by M-mode at the lateral tricuspid annulus in the apical four-chamber view; in accordance with current guidelines, TAPSE < 17 mm was defined as right ventricular systolic dysfunction. Pulmonary artery pressure (PAP) was also measured to evaluate left ventricular diastolic dysfunction and right ventricular afterload, and the values were recorded.

Following completion of the echocardiographic examination, a 24-h ambulatory blood pressure monitoring (ABPM) device was applied to each patient on the same day, and measurements were initiated in accordance with standard protocols.

### 2.4. Ambulatory Blood Pressure Monitoring (ABPM)

ABPM was performed using validated oscillometric device (TONOPORT V) in accordance with international guidelines. Measurements were obtained every 20 min during the daytime (06:00–22:00) and every 30–60 min during the nighttime (22:00–06:00). Patients were instructed to record their sleep and wake times and to continue their usual daily activities and antihypertensive medications. In accordance with the ESC 2024 guidelines, an ABPM recording was considered valid if at least 70% of the measurements were usable.

Morning blood pressure was defined, in line with the ESC 2024 guidelines, as the average systolic and diastolic blood pressure values recorded within the first two hours after awakening. Morning hypertension was defined as ≥135/85 mmHg, while nocturnal hypertension was defined using a threshold of ≥120/70 mmHg. Dipping patterns were classified based on the ratio of nighttime to daytime systolic and diastolic blood pressure: values between 0.8–0.9 were considered dipper, 0.9–1.0 non-dipper, and >1.0 reverse dipper. Morning hypertension, nocturnal hypertension, and dipping status were determined according to these classifications.

### 2.5. Ethical Approval

The study protocol was approved by the Clinical Research Ethics Committee of Giresun Training and Research Hospital (Application No: KAEK 161). The project was deemed ethically appropriate at the meeting held on 11 September 2023, with decision number 11.09.2023/02. Institutional permission was obtained prior to data collection. The study was conducted in accordance with the principles of the Declaration of Helsinki.

### 2.6. Statistical Analysis

Statistical analyses were performed using IBM SPSS Statistics version 31.0 and AMOS 31 software packages. The sample size was determined based on the number of eligible CKD patients presenting during the study period, reflecting a feasibility-based approach. Continuous variables were expressed as mean ± standard deviation or median (minimum–maximum), as appropriate, and categorical variables were presented as number (*n*) and percentage (%). Normality of continuous variables was evaluated using skewness and kurtosis values (±1.96). Variables with normal distribution were analyzed using the independent samples *t*-test, whereas non-normally distributed variables were compared using the Mann–Whitney U test. Categorical variables were assessed using the chi-square test. Spearman correlation analysis was chosen because several variables did not meet parametric assumptions. Regression models included variables that were clinically relevant and significant in univariable analyses, and assumptions of linearity and multicollinearity were checked to ensure model validity. Correlation coefficients (r) and significance levels were reported. Correlation strength was interpreted as follows: 0.00–0.30 low, 0.30–0.70 moderate, and 0.70–1.00 high. Correlation analyses were exploratory; therefore, no formal correction for multiple comparisons was applied. A correlation matrix heatmap with a red–blue color scale was generated to visually illustrate the correlation results. Variables included in the regression models were selected based on clinical relevance and significance in univariable analyses, in order to avoid overadjustment while maintaining model robustness. Multivariable models were constructed using a parsimonious, clinically prespecified approach that incorporated key covariates known to influence renal function (morning and nocturnal hypertension). Sex, diabetes mellitus, and antihypertensive treatment were not included in the final model because these variables did not differ significantly between groups and introduced multicollinearity when added to preliminary models. Path analysis was performed to examine the effects of morning and nocturnal hypertension on renal and cardiac target organ damage. A *p*-value < 0.05 was considered statistically significant. Sex and diabetes mellitus were evaluated in preliminary regression models; however, their inclusion resulted in increased variance inflation and reduced model stability due to strong biological associations with renal function parameters. Therefore, a parsimonious, clinically prespecified modeling strategy was adopted to avoid overadjustment. Sensitivity assessments performed in preliminary models showed that the direction and significance of the association between morning hypertension and eGFR remained unchanged when sex and diabetes were added.

## 3. Results

Among the 132 hypertensive patients with chronic kidney disease included in the study, 42.4% had morning hypertension (Table 1). There were no significant differences between the groups in terms of age, sex, or diabetes mellitus. However, the prevalence of coronary artery disease was significantly higher in patients with morning hypertension (25.0% vs. 9.2%; *p* = 0.027). Office hypertension and nocturnal hypertension were also more common in the morning hypertension group (*p* = 0.022 and *p* < 0.001, respectively).

In the echocardiographic assessment, TAPSE values were significantly lower in patients with morning hypertension (*p* = 0.031). No significant differences were observed between the groups regarding LVEF, IVSd, or PAP. Evaluation of renal function parameters revealed that patients with morning hypertension had significantly lower eGFR (*p* < 0.001) and significantly higher UACR levels (*p* < 0.001).

Nocturnal hypertension was present in 78.0% of the patients (Table 2). Patients with nocturnal hypertension more frequently exhibited office hypertension (*p* < 0.001) and had a lower prevalence of the dipper pattern (*p* = 0.010). IVSd values and the frequency of increased IVSd were significantly higher in the nocturnal hypertension group (*p* = 0.033 and *p* = 0.016, respectively). UACR levels were also higher among patients with nocturnal hypertension (*p* = 0.003). No significant differences were observed in other clinical or echocardiographic parameters.

Office hypertension was identified in 59.1% of the patients (Table 3). Patients with office hypertension were older (*p* = 0.044), and the use of renin–angiotensin–aldosterone system (RAAS) blockers was more common in those without office hypertension (*p* = 0.030). The relatively low use of RAAS blockers in our cohort may be related to the high proportion of patients with advanced CKD, in whom hyperkalemia and other adverse effects can limit RAAS inhibitor use; however, this explanation should be interpreted with caution, as specific reasons for non-use were not systematically documented. No significant differences were found in echocardiographic or renal parameters.

In the correlation analysis (Table 4), morning hypertension showed positive correlations with coronary artery disease, urea, creatinine, UACR, PTH, and erythrocyte sedimentation rate, and negative correlations with eGFR, albumin, calcium, hemoglobin, platelet count, and TAPSE (*p* < 0.05). Nocturnal hypertension was positively correlated with IVSd and UACR, and negatively correlated with the dipper pattern and albumin. Office hypertension was positively associated only with age and negatively associated with RAAS blocker use.

In the multivariable regression analysis (Table 5), morning hypertension was found to have an independent and negative effect on eGFR (β = −0.26; *p* = 0.002). Nocturnal hypertension demonstrated an independent and positive effect on IVSd (β = 0.19; *p* = 0.024). Neither morning nor nocturnal hypertension showed an independent association with TAPSE or UACR. Age was negatively associated with eGFR (*p* = 0.044), and coronary artery disease had a negative effect on TAPSE (*p* = 0.025).

## 4. Discussion

In this cross-sectional study, morning hypertension was shown to be associated with renal and cardiac target organ damage in individuals with hypertension and chronic kidney disease. In the initial analyses, morning hypertension was associated with lower eGFR, higher UACR levels, and reduced TAPSE values, reflecting impaired right ventricular systolic function. This reduction in right ventricular function may be related to increased systemic hemodynamic stress or pulmonary vascular loading during the early morning hours. However, in the multivariable regression analysis, the association between morning hypertension and UACR was no longer independent, whereas morning hypertension remained independently associated with lower eGFR. These findings suggest that morning blood pressure levels may be clinically relevant in CKD and indicate that morning hypertension could represent a distinct blood pressure phenotype in this patient population.

Our findings are consistent with previous studies demonstrating the association of morning hypertension with both renal and cardiac structural alterations. In a large cohort of 447 CKD patients, Liu et al. reported that morning hypertension was associated with lower eGFR, higher left ventricular mass index (LVMI), increased proteinuria, and a two-fold higher risk of LVH, independent of nocturnal hypertension and the morning blood pressure surge [6]. Similar studies have also shown that morning hypertension is strongly associated with cardiac damage and impaired kidney function in patients with CKD [13,14].

The existing literature on the clinical significance of morning hypertension in CKD remains quite limited. In the systematic review published by Faiz et al. in 2026, only two cohort studies were identified that demonstrated an independent association between the morning blood pressure surge (MBPS) and renal as well as composite clinical outcomes, highlighting that morning blood pressure patterns have not been adequately investigated in CKD populations [15]. Furthermore, a large cohort study published in *Nutrition, Metabolism & Cardiovascular Diseases* in 2022 reported a significant association between morning hypertension and adverse renal outcomes. These studies support the link between morning hypertension and renal target organ damage; however, most investigations have focused solely on MBPS and have not evaluated cardiac parameters (IVSd, TAPSE) or incorporated analytical models that simultaneously assess both morning and nocturnal hypertension [14]. In this context, our study contributes to the literature by evaluating both renal (eGFR, UACR) and cardiac (IVSd, TAPSE) target organ damage concurrently and by demonstrating that morning hypertension is independently associated with lower eGFR.

The associations of morning hypertension with UACR, creatinine, and inflammatory markers suggest that elevated morning blood pressure may contribute to renal injury through both hemodynamic and non-hemodynamic mechanisms. However, the lack of independent associations for these parameters in multivariable analyses may be related to the limited sample size and the reduced statistical power inherent to the cross-sectional design of our study. In our study, nocturnal hypertension was highly prevalent, consistent with the existing literature, and was independently associated with increased IVSd. This finding supports the substantial impact of nighttime blood pressure on cardiac remodeling in CKD. Previous studies have shown that non-dipping and nocturnal hypertension occur in approximately 80% of patients with CKD and are associated with an increased risk of LVH, proteinuria, and cardiovascular events [13,16,17]. Earlier research has also emphasized the superiority of ABPM over office measurements in assessing both target organ damage and clinical risk in CKD [18,19]. Our results further support this view by demonstrating the association of nocturnal hypertension with IVSd and UACR in CKD patients. A substantial overlap between morning and nocturnal hypertension was observed in our cohort, as 92.9% of patients with morning hypertension also had nocturnal hypertension. Therefore, morning hypertension should not necessarily be interpreted as a completely distinct blood pressure phenotype in this population. Although morning hypertension remained independently associated with eGFR after adjustment for nocturnal hypertension, the considerable coexistence of these ABPM-derived phenotypes warrants cautious interpretation of this association.

The high coexistence of morning and nocturnal hypertension suggests that these two phenotypes may share partially overlapping yet distinct pathophysiological mechanisms. Increased sympathetic activation, sodium retention, endothelial dysfunction, and impaired circadian rhythm—common features in CKD—may contribute to both phenotypes. Morning hypertension, in particular, may be related to reduced antihypertensive drug efficacy in the early morning hours, inadequate nocturnal blood pressure control, or heightened morning sympathetic activity. In our study, the associations of morning hypertension with inflammation (erythrocyte sedimentation rate), mineral metabolism (PTH, calcium), and anemia (hemoglobin) suggest that this phenotype may have a multifaceted pathophysiological basis.

The independent effect of morning hypertension on eGFR in the multivariable analyses is particularly noteworthy. Given the relatively small sample size, these associations should be considered exploratory and hypothesis-generating rather than definitive. Given the progressive nature of CKD, early identification of patients with morning hypertension may contribute to the development of more targeted therapeutic strategies aimed at slowing the decline in kidney function.

The limited association of office blood pressure measurements with target organ damage indicates that relying solely on office readings may be insufficient for monitoring patients with CKD. In the CRIC cohort, Thomas et al. reported that blood pressure patterns identified by ABPM predicted clinical outcomes more accurately than office measurements [20]. Importantly, ABPM-derived phenotypes showed associations with renal and cardiac parameters that were largely absent for Office hypertension. In particular, morning hypertension was independently associated with lower eGFR, whereas nocturnal hypertension was associated with increased IVSd, highlighting the additional information provided by ABPM beyond Office blood pressure measurements. Additionally, the associations of nocturnal hypertension with IVSd and UACR provide further evidence supporting the clinical value of ABPM in assessing target organ damage in CKD.

Among the strengths of our study are the comprehensive ABPM assessment, the detailed echocardiographic evaluation, and the use of multivariable models designed to distinguish the independent effects of morning and nocturnal hypertension. However, the cross-sectional design does not allow for the determination of causal relationships. Longitudinal studies are needed to evaluate whether morning hypertension directly contributes to renal and cardiac damage. The modest sample size substantially limits statistical power and restricts the generalizability of our findings. Therefore, all associations observed in this study should be interpreted with caution. Furthermore, the modest explanatory power of the multivariable models, including an R^2^ of 0.10 for eGFR, indicates that these models should not be interpreted as tools for individual risk prediction. The inclusion of a broad spectrum of CKD stages, ranging from stage 1 to stage 5, may also have complicated the interpretation of the effects of morning hypertension on renal and cardiac target organ damage. In particular, the small number of patients with early-stage CKD reduced the interpretability of subgroup analyses. BMI, smoking status, and HbA1c levels were not included in the analysis because these variables were not consistently available for all participants, and inclusion of incomplete data could introduce bias. It should also be noted that the timing of antihypertensive medication intake may influence morning and nocturnal blood pressure profiles; however, dosing times were not systematically recorded in our cohort and therefore could not be included in the analysis. Future studies incorporating precise medication timing data may provide additional insights into these relationships. Taken together, these limitations indicate that morning hypertension should be interpreted as a potential early marker rather than a definitive indicator of renal dysfunction, and ABPM-based risk stratification should be applied cautiously in heterogeneous CKD populations. Given the cross-sectional design and modest explanatory power of the models, statements regarding risk stratification and individualized treatment should be interpreted cautiously.

Overall, our findings indicate that morning hypertension represents a clinically important blood pressure phenotype in CKD. Morning hypertension is associated with both renal and cardiac target organ damage, and its association with lower eGFR may help identify patients with renal involvement; however, longitudinal studies are required to determine whether it has prognostic significance. Given the high prevalence of both morning and nocturnal hypertension in CKD, incorporating ABPM into clinical evaluation may provide additional information on blood pressure phenotypes and their associations with target-organ involvement beyond that obtained from Office blood pressure measurements alone.

## 5. Conclusions

In conclusion, our study suggests that morning hypertension may represent a clinically important blood pressure phenotype in chronic kidney disease; however, given the modest sample size, these findings should be interpreted as exploratory rather than definitive. The independent association between morning hypertension and eGFR suggests that this phenotype may be related to renal involvement, but larger studies are needed to clarify its prognostic significance. The associations of nocturnal hypertension with IVSd and UACR further support the importance of evaluating 24-h blood pressure patterns in assessing target organ damage in CKD. Taken together, these findings suggest that ABPM-derived blood pressure phenotypes may provide clinically relevant information on renal and cardiac target-organ involvement that is not captured by office blood pressure measurements alone. Nevertheless, prospective studies are required to establish causality and to determine the long-term renal and cardiovascular consequences of morning hypertension.

## Figures and Tables

**Table 1:** Comparison of Demographic, Clinical, Echocardiographic, and Renal Characteristics of Chronic Kidney Disease Patients with and Without Morning Hypertension.

Variable	Group	Overall *n* (%)	No Morning HT *n* (%)	Morning HT *n* (%)	*p*
Sex	Male	76 (57.6)	44 (57.9)	32 (57.1)	1.000
	Female	56 (42.4)	32 (42.1)	24 (42.9)	
Coronary artery disease	No	111 (84.1)	69 (90.8)	42 (75.0)	0.027 *
	Yes	21 (15.9)	7 (9.2)	14 (25.0)	
Diabetes mellitus	No	74 (56.1)	44 (57.9)	30 (53.6)	0.751
	Yes	58 (43.9)	32 (42.1)	26 (46.4)	
Hypertension	No	7 (5.3)	5 (6.6)	2 (3.6)	0.712
	Yes	125 (94.7)	71 (93.4)	54 (96.4)	
Heart failure	No	130 (98.5)	76 (100.0)	54 (96.4)	0.348
	Yes	2 (1.5)	0 (0.0)	2 (3.6)	
RAAS blocker use	No	106 (80.3)	63 (82.9)	43 (76.8)	0.515
	Yes	26 (19.7)	13 (17.1)	13 (23.2)	
Calcium channel blocker	No	110 (83.3)	63 (82.9)	47 (83.9)	1.000
	Yes	22 (16.7)	13 (17.1)	9 (16.1)	
Combination antihypertensive therapy	No	50 (37.9)	27 (35.5)	23 (41.1)	0.640
	Yes	82 (62.1)	49 (64.5)	33 (58.9)	
Office hypertension	No	54 (40.9)	38 (50.0)	16 (28.6)	0.022 *
	Yes	78 (59.1)	38 (50.0)	40 (71.4)	
Nocturnal hypertension	No	29 (22.0)	25 (32.9)	4 (7.1)	<0.001 *
	Yes	103 (78.0)	51 (67.1)	52 (92.9)	
Dipper pattern	No	113 (85.6)	66 (86.8)	47 (83.9)	0.826
	Yes	19 (14.4)	10 (13.2)	9 (16.1)	
IVSd	Normal (<1.2 cm)	92 (69.7)	56 (73.7)	36 (64.3)	0.332
	Increased (≥1.2 cm)	40 (30.3)	20 (26.3)	20 (35.7)	
TAPSE	Low (<1.7 cm)	58 (43.9)	27 (35.5)	31 (55.4)	0.036 *
	Normal (≥1.7 cm)	74 (56.1)	49 (64.5)	25 (44.6)	
	Overall Mean ± SD, Median (Min–Max)		No Morning HT Mean ± SD, Median (Min–Max)	Morning HT Mean ± SD, Median (Min–Max)	*p*
Age (years)	64.88 ± 10.04; 66.5 (35–80)		63.99 ± 9,92; 64.5 (35–80)	66.11 ± 10.17; 68 (44–80)	0.232
LVEF (%)	63.11 ± 4.43; 65 (35–65)		63.45 ± 3.37; 65 (50–65)	62.64 ± 5.56; 65 (35–65)	0.449
IVSd	1.07 ± 0.15; 1.10 (0.70–1.50)		1.06 ± 0.15; 1.10 (0.70–1.50)	1.08 ± 0.16; 1.10 (0.70–1.40)	0.562
TAPSE (cm)	1.71 ± 0.24; 1.70 (1.00–2.70)		1.73 ± 0.22; 1.70 (1.30–2.70)	1.67 ± 0.26; 1.60 (1.00–2.40)	0.031 *
PAP (mmHg)	25.94 ± 7.93; 25 (11–50)		25.75 ± 7.82; 25 (12–50)	26.20 ± 8.17; 25 (11–45)	0.771
eGFR (mL/min/1.73 m^2^)	40.79 ± 16.17; 40.4 (6.2–87.4)		44.78 ± 14.44; 47.4 (6.2–80.0)	35.37 ± 16.92; 31.9 (9.8–87.4)	<0.001 *
UACR (mg/g)	497.48 ± 1039.28; 106.8 (1.7–7273)		344.74 ± 971.31; 39.35 (1.7–7273)	704.77 ± 1100.04; 263.25 (2.7–6376)	<0.001 *

Mean: Mean; SD: Standard deviation; Med: Median; Min: Minimum; Max: Maximum. Categorical variables were compared using the chi-square (χ^2^) test. Continuous variables were compared using the independent samples *t*-test (t) or the Mann–Whitney U test (z), depending on distribution. Statistical significance was accepted as *p* < 0.05. * indicates statistical significance.

**Table 2:** Comparison of Demographic, Clinical, Echocardiographic, and Renal Characteristics of Chronic Kidney Disease Patients with and Without Nocturnal Hypertension.

Variable	Group	No Nocturnal HT *n* (%)	Nocturnal HT *n* (%)	*p*
Sex	Male	19 (65.5)	57 (55.3)	0.443
	Female	10 (34.5)	46 (44.7)	
Coronary artery disease	No	25 (86.2)	86 (83.5)	0.948
	Yes	4 (13.8)	17 (16.5)	
Diabetes mellitus	No	19 (65.5)	55 (53.4)	0.342
	Yes	10 (34.5)	48 (46.6)	
Hypertension	No	1 (3.4)	6 (5.8)	0.972
	Yes	28 (96.6)	97 (94.2)	
Heart failure	No	29 (100.0)	101 (98.1)	1.000
	Yes	0 (0.0)	2 (1.9)	
RAAS blocker use	No	24 (82.8)	82 (79.6)	0.911
	Yes	5 (17.2)	21 (20.4)	
Calcium channel blocker	No	23 (79.3)	87 (84.5)	0.707
	Yes	6 (20.7)	16 (15.5)	
Combination antihypertensive therapy	No	11 (37.9)	39 (37.9)	1.000
	Yes	18 (62.1)	64 (62.1)	
Office hypertension	No	20 (69.0)	34 (33.0)	<0.001 *
	Yes	9 (31.0)	69 (67.0)	
Dipper pattern	No	20 (69.0)	93 (90.3)	0.010 *
	Yes	9 (31.0)	10 (9.7)	
IVSd	Normal (<1.2 cm)	26 (89.7)	66 (64.1)	0.016 *
	Increased (≥1.2 cm)	3 (10.3)	37 (35.9)	
TAPSE	Low (<1.7 cm)	11 (37.9)	47 (45.6)	0.599
	Normal (≥1.7 cm)	18 (62.1)	56 (54.4)	
Variable	No Nocturnal HT Mean ± SD, Median (Min–Max)		Nocturnal HT Mean ± SD, Median (Min–Max)	*p*
Age (years)	63.93 ± 8.48; 63 (41–79)		65.16 ± 10.46; 68 (35–80)	0.564
LVEF (%)	63.79 ± 2.88; 65 (55–65)		62.91 ± 4.77; 65 (35–65)	0.315
IVSd	1.02 ± 0.14; 1.00 (0.80–1.40)		1.09 ± 0.16; 1.10 (0.70–1.50)	0.033 *
TAPSE (cm)	1.73 ± 0.18; 1.80 (1.40–2.20)		1.70 ± 0.26; 1.70 (1.00–2.70)	0.176
PAP (mmHg)	26.43 ± 7.74; 25 (12–40)		25.80 ± 8.02; 25 (11–50)	0.735
eGFR (mL/min/1.73 m^2^)	43.48 ± 13.73; 47.4 (11.1–65.0)		40.03 ± 16.77; 39.4 (6.2–87.4)	0.312
UACR (mg/g)	190.49 ± 354.34; 20 (1.7–1430)		583.92 ± 1148.20; 124.5 (2.7–7273)	0.003 *

Mean: Mean; SD: Standard deviation; Med: Median; Min: Minimum; Max: Maximum. Categorical variables were compared using the chi-square (χ^2^) test. Continuous variables were compared using the independent samples *t*-test (t) or the Mann–Whitney U test (z), depending on distribution. Statistical significance was accepted as *p* < 0.05. * indicates statistical significance.

**Table 3:** Comparison of Demographic, Clinical, Echocardiographic, and Renal Characteristics of Chronic Kidney Disease Patients with and Without Office Hypertension.

Variable	Group	No Office HT *n* (%)	Office HT *n* (%)	*p*
Sex	Male	30 (55.6)	46 (59.0)	0.832
	Female	24 (44.4)	32 (41.0)	
Coronary artery disease	No	46 (85.2)	65 (83.3)	0.965
	Yes	8 (14.8)	13 (16.7)	
Diabetes mellitus	No	32 (59.3)	42 (53.8)	0.662
	Yes	22 (40.7)	36 (46.2)	
Hypertension	No	3 (5.6)	4 (5.1)	1.000
	Yes	51 (94.4)	74 (94.9)	
Heart failure	No	54 (100.0)	76 (97.4)	0.645
	Yes	0 (0.0)	2 (2.6)	
RAAS blocker use	No	38 (70.4)	68 (87.2)	0.030 *
	Yes	16 (29.6)	10 (12.8)	
Calcium channel blocker	No	47 (87.0)	63 (80.8)	0.476
	Yes	7 (13.0)	15 (19.2)	
Combination antihypertensive therapy	No	24 (44.4)	26 (33.3)	0.266
	Yes	30 (55.6)	52 (66.7)	
Dipper pattern	No	44 (81.5)	69 (88.5)	0.384
	Yes	10 (18.5)	9 (11.5)	
IVSd	Normal (<1.2 cm)	38 (70.4)	54 (69.2)	1.000
	Increased (≥1.2 cm)	16 (29.6)	24 (30.8)	
TAPSE	Low (<1.7 cm)	23 (42.6)	35 (44.9)	0.935
	Normal (≥1.7 cm)	31 (57.4)	43 (55.1)	
Variable	No Office HT Mean ± SD, Median (Min–Max)		Office HT Mean ± SD, Median (Min–Max)	*p*
Age (years)	62.78 ± 10.21; 64 (41–80)		66.35 ± 9.72; 68 (35–80)	0.044 *
LVEF (%)	62.30 ± 6.02; 65 (35–65)		63.67 ± 2.77; 65 (54–65)	0.284
IVSd	1.06 ± 0.16; 1.10 (0.70–1.50)		1.08 ± 0.15; 1.10 (0.70–1.40)	0.612
TAPSE (cm)	1.69 ± 0.24; 1.70 (1.00–2.40)		1.72 ± 0.25; 1.70 (1.40–2.70)	0.970
PAP (mmHg)	26.44 ± 9.54; 25 (11–50)		25.60 ± 6.72; 25 (12–40)	0.592
eGFR (mL/min/1.73 m^2^)	41.33 ± 15.04; 40 (6.2–75.0)		40.41 ± 16.99; 41.65 (9.8–87.4)	0.751
UACR (mg/g)	343.44 ± 700.04; 38.05 (1.7–3870)		604.12 ± 1213.30; 125 (2.3–7273)	0.067

Mean: Mean; SD: Standard deviation; Med: Median; Min: Minimum; Max: Maximum. Categorical variables were compared using the chi-square (χ^2^) test. Continuous variables were compared using the independent samples *t*-test (t) or the Mann–Whitney U test (z), depending on distribution. Statistical significance was accepted as *p* < 0.05. * indicates statistical significance.

**Table 4:** Correlation Analysis Between Morning, Office, and Nocturnal Hypertension and Demographic, Clinical, Echocardiographic, and Renal Parameters.

Variable	Morning HT r	Morning HT *p*	Office HT r	Office HT *p*	Nocturnal HT r	Nocturnal HT *p*
Sex	0.008	0.932	−0.034	0.699	0.085	0.331
Age	0.120	0.170	0.172 *	0.049	0.089	0.308
Coronary artery disease	0.213 *	0.014	0.025	0.777	0.031	0.727
Diabetes mellitus	0.043	0.624	0.054	0.541	0.101	0.249
Hypertension	0.066	0.450	0.009	0.915	−0.044	0.617
RAS blocker use	0.076	0.387	−0.208 *	0.017	0.033	0.709
Calcium channel blocker use	−0.014	0.876	0.083	0.346	−0.057	0.514
Combination antihypertensive therapy	−0.056	0.520	0.113	0.199	0.001	0.995
Dipper pattern	0.041	0.640	−0.098	0.265	−0.252 *	0.004
LVEF	−0.066	0.451	0.094	0.285	−0.088	0.317
IVSd	0.047	0.592	0.060	0.496	0.217 *	0.013
TAPSE	−0.189 *	0.030	0.003	0.970	−0.118	0.177
Pulmonary artery pressure	0.036	0.712	−0.012	0.902	−0.051	0.603
Urea	0.228 *	0.009	−0.009	0.916	0.062	0.480
Creatinine	0.309 *	<0.001	0.003	0.970	0.097	0.269
eGFR	−0.327 *	<0.001	−0.038	0.665	−0.120	0.171
UACR	0.288 *	<0.001	0.160	0.067	0.262 *	0.002
Total cholesterol	−0.052	0.551	0.023	0.789	0.012	0.887
HDL	0.091	0.298	0.061	0.486	0.078	0.375
LDL	−0.020	0.821	−0.040	0.645	−0.036	0.680
Albumin	−0.281 *	0.001	−0.126	0.150	−0.292 *	<0.001
Calcium	−0.249 *	0.004	−0.092	0.292	−0.101	0.248
Phosphorus	0.093	0.290	−0.023	0.791	0.109	0.215
PTH	0.336 *	<0.001	0.029	0.739	0.088	0.316
ESR	0.241 *	0.005	0.082	0.349	0.010	0.909
Hemoglobin	−0.215 *	0.013	−0.010	0.910	−0.077	0.383
Platelet count	−0.192 *	0.028	−0.040	0.650	−0.155	0.076

Spearman correlation analysis was used. * Indicates statistical significance at *p* < 0.05.

**Table 5:** Multivariable Regression Analysis Evaluating the Effects of Morning and Nocturnal Hypertension on Renal and Cardiac Target Organ Damage in Chronic Kidney Disease.

Dependent Variable	Path	Independent Variable	B	S.E.	β (Beta)	*p*	R^2^
UACR	←	Age	−7.77	8.81	−0.08	0.378	0.046
	←	Morning HT	324.45	178.37	0.16	0.069	
	←	Coronary artery disease	−139.51	241.02	−0.05	0.563	
	←	Nocturnal HT	287.68	212.92	0.12	0.177	
GFR	←	Age	−0.27	0.13	−0.17	0.044 *	0.100
	←	Morning HT	−8.49	2.68	−0.26	0.002 *	
	←	Coronary artery disease	−2.38	3.62	−0.06	0.511	
	←	Nocturnal HT	0.06	3.20	0.00	0.986	
IVSd	←	Morning HT	−0.01	0.03	−0.03	0.707	0.049
	←	Nocturnal HT	0.07	0.03	0.19	0.024 *	
	←	Age	0.00	0.00	0.01	0.910	
	←	Coronary artery disease	0.05	0.04	0.11	0.206	
TAPSE	←	Age	0.00	0.00	0.07	0.430	0.050
	←	Morning HT	−0.04	0.04	−0.09	0.285	
	←	Nocturnal HT	−0.01	0.05	−0.01	0.872	
	←	Coronary artery disease	−0.13	0.06	−0.19	0.025 *	

* Indicates statistical significance at *p* < 0.05. B: Unstandardized regression coefficient; S.E.: Standard error; β: Standardized regression coefficient; R^2^: Explained variance.

## Data Availability

The data supporting the findings of this study are available from the corresponding author upon reasonable request. Due to institutional regulations and patient privacy, raw data cannot be publicly shared.

## Abbreviations

CKD	Chronic kidney disease
ABPM	Ambulatory blood pressure monitoring
LVH	Left ventricular hypertrophy
UACR	Urine albumin-to-creatinine ratio
eGFR	Estimated glomerular filtration rate
IVSd	Interventricular septal thickness
TAPSE	Tricuspid annular plane systolic excursion
